# Evaluation of Obesity Influence in the Sexual Function of Postmenopausal Women: A Cross-Sectional Study

**DOI:** 10.1055/s-0039-1700795

**Published:** 2019-11

**Authors:** Gustavo Maximiliano Dutra da Silva, Sônia Maria Rolim Rosa Lima, Benedito Fabiano dos Reis, Carolina Furtado Macruz, Sóstenes Postigo

**Affiliations:** 1 Department of Obstetrics and Gynecology, Santa Casa de Misericórdia de São Paulo, São Paulo, SP, Brazil; 2 Universidade São Francisco, Bragança Paulista, SP, Brazil; 3 Departamento de Ginecologia e Obstetrícia, Universidade do Vale do Sapucaí, Pouso Alegre, MG, Brazil

**Keywords:** obesity, metabolic syndrome, body mass index, menopause, female sexual dysfunction, obesidade, síndrome metabólica, índice de massa corporal, menopausa, disfunção sexual feminina

## Abstract

**Objective** The incidence of obesity, which is a chronic condition, has increased in recent years. The association between obesity and female sexual dysfunction remains unclear, particularly in postmenopausal women. In the present study, we evaluated whether obesity is a risk factor for sexual dysfunction in postmenopausal women.

**Methods** This is a cross-sectional study that analyzed data from interviews of postmenopausal women at the Climacteric Outpatient Clinic from 2015 to 2018. After applying the inclusion and exclusion criteria, 221 women aged between 40 and 65 years old were selected and invited to participate in the study. Obesity was diagnosed according to body mass index (BMI). The participants were grouped into the following BMI categories: group 1, 18.5–24.9 kg/m^2^ (normal); group 2, 25.0–29.9 kg/m^2^ (overweight); and group 3, ≥30.0 kg/m^2^ (obese). Sexual function was assessed using the Female Sexual Function Index (FSFI) questionnaire. Cutoff points of ≥23 and ≥26.5 were adopted to define a diagnosis of female sexual dysfunction (FSD) based on the Diagnostic and Statistical Manual of Mental Disorders, 4^th^ edition, Text Revision by the American Psychiatric Association (DSM-IV-TR).

**Results** The desire and arousal scores were statistically higher in the normal BMI group than in the obese group (*p* = 0.028 and *p* = 0.043, respectively). The satisfaction scores were statistically higher in the normal BMI group than in the overweight and obese groups (*p* < 0.05). The total FSFI score statistically differed among the BMI categories (*p* = 0.027).

**Conclusion** In the present study, obese and overweight postmenopausal women had higher total scores than women with normal BMI. Our results show that obese and overweight postmenopausal women had a higher index of dysfunction in desire and arousal and lower sexual satisfaction than normal-weight women.

## Resumo

**Objetivo** A incidência de obesidade, que é uma condição crônica, aumentou nos últimos anos. A associação entre obesidade e disfunção sexual feminina ainda não está clara, particularmente em mulheres após a menopausa. No presente estudo, avaliamos se a obesidade é um fator de risco para disfunção sexual em mulheres após a menopausa.

**Métodos** Este é um estudo transversal que analisou dados de entrevistas de mulheres após a menopausa no Ambulatório de Climatério a partir de 2015 até 2018. Após a aplicação dos critérios de inclusão e exclusão, 221 mulheres com idade entre 40 e 65 anos foram selecionadas e convidadas a participar do estudo. Obesidade foi diagnosticada de acordo com o índice de massa corpórea (IMC). Os participantes foram agrupados nas seguintes categorias de IMC: grupo 1: 18,5–24,9 kg/m2 (normal); grupo 2: 25,0–29,9 kg/m2 (sobrepeso); e grupo 3: ≥ 30,0 kg/m2 (obesidade). A função sexual foi avaliada através do questionário Female Sexual Function Index (FSFI, na sigla em inglês). Pontos de corte de ≥ 23 e ≥ 26,5 foram adotados para definir um diagnóstico de disfunção sexual feminina (DSF) com base no Manual de Diagnóstico e Estatística das Perturbações Mentais, 4ª Edição, Texto Revisto DSM-IV-TR.

**Resultados** Os escores de desejo e excitação foram estatisticamente maiores no grupo com IMC normal do que no grupo obesidade (*p* = 0,028 e *p* = 0,043, respectivamente). Os escores de satisfação foram estatisticamente maiores no grupo com IMC normal do que nos grupos com sobrepeso e obesidade (*p* < 0,05). A pontuação total do FSFI diferiu estatisticamente entre as categorias de IMC (*p* = 0,027).

**Conclusão** No presente estudo, mulheres após a menopausa obesas e com sobrepeso tiveram escores totais mais altos do que mulheres com IMC normal. Nossos resultados mostram que mulheres obesas e com sobrepeso após a menopausa apresentaram índices mais altos de disfunção no desejo e excitação e menor satisfação sexual do que mulheres com peso normal.

## Introduction

Obesity is a chronic condition with an increased incidence in recent years, both in developed and developing countries. The World Health Organization (WHO) estimates > 1 billion people to be overweight, with ∼ 300 million meeting the criteria for obesity. By 2025, 2.3 billion and 700 million adults are predicted to be overweight and obese, respectively.^1^

Data from the Family Budget Survey (POF, in the Portuguese acronym)^2^ show a steady increase in the incidence of overweight and obesity in Brazilians aged > 20 years. Data from the 2008–2009 POF were compared with surveys conducted in 1974–1975 (National Study of Family Expenditure), in 1989 (National Survey on Health and Nutrition), and the 2002–2003 POF. The results showed that over the 35-year period, the prevalence of overweight and obesity in women increased from 28.7% to 48% and from 8% to 16.9% (an almost 2-fold increase), respectively. The combined prevalence of overweight and obesity in women aged between 45 and 54 years old was 58%, whereas among women aged between 55 and 64 years old, it was 63%. Each person requires daily energy for basal metabolism, food digestion, and physical activity. Obesity results from an imbalance between energy expenditure and caloric intake and has a multifactorial etiology. In the development of obesity, genetics is an important predisposing factor that can interact with environmental factors.^3^

The association between obesity and menopause has been the focus of numerous studies. During menopause, a shift in fat redistribution occurs toward the central (android) type.^3^ ^4^ ^5^ This type of distribution is often associated with systemic arterial hypertension (SAH), insulin resistance and hyperinsulinism, glucose intolerance, hypertriglyceridemia, decreased serum levels of high-density lipoprotein (HDL) cholesterol and apolipoprotein A1, and changes in fibrinolysis. Collectively, these changes were first described by Reaven as X syndrome, but it is currently referred to as metabolic syndrome (MetS).^6^ ^7^ ^8^ ^9^

Female sexual dysfunction (FSD) is a condition affecting women of different ages and ethnicities. Female sexual dysfunction is characterized by psychophysiological disturbances and abnormalities in the sexual response cycle, including disturbances in sexual desire and orgasm accompanied by pain. This dysfunction is highly prevalent and is influenced by organic and psychosocial health-related factors.^10^ The significant association of FSD and older age among different ethnic groups, which is maintained in the regression model, is supported by several previous studies.^11^ ^12^ ^13^

Sexual function in obese women has only recently come under study, and researchers have highlighted the scarcity of studies investigating female sexuality. Thus, there is a need for routine assessment of sexual function in women with obesity, overweight, and MetS, together with the respective complications and prevalence of these conditions and their impact on quality of life.^14^ Given the high prevalence of obesity with aging and the serious health repercussions of being overweight, we sought to investigate obesity, and its relationship with FSD.

The present study aimed to evaluate whether obesity is a risk factor for FSD in postmenopausal women.

## Methods

**Fig. 1:**
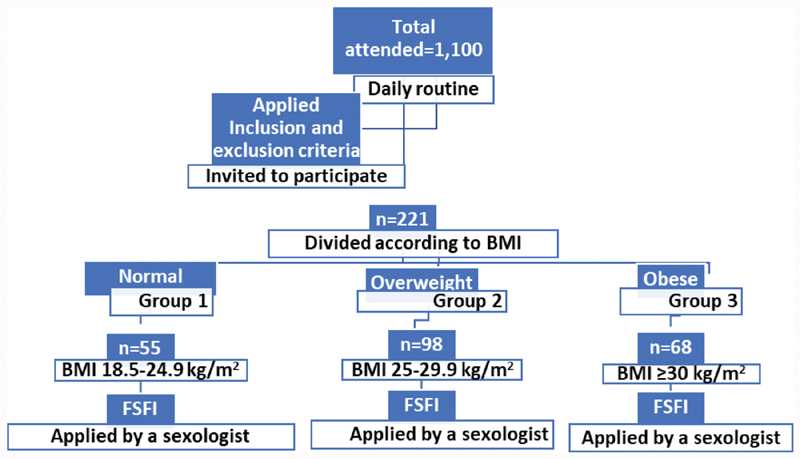
Flow of the study in which postmenopausal women were stratified according to body mass index (BMI). Sexual function was evaluated using the Female Sexual Function Index (FSFI) questionnaire.^3^ **Source:** Santos^3^

In this cross-sectional study, we analyzed data from interviews of 1,100 postmenopausal women at the Climacteric Outpatient Clinic of the School of Medical Sciences of the Hospital Santa Casa de Misericórdia de São Paulo and at the Care Management Unit IV of the Hospital Maternidade Leonor Mendes de Barros from 2015 to 2018. Women who regularly visit these institutions to undergo routine climacteric examinations were invited to participate in the study after their examinations. After applying the inclusion and exclusion criteria, 221 women were selected and invited to participate in the study (Fig. 1).^3^

Women who regularly visit these institutions to undergo routine climacteric examinations were invited to participate in the study after their examinations. The present work is an early result of a doctoral thesis developed at the Faculdade de Ciências Médicas of the Hospital Santa Casa de Misericórdia de São Paulo, which intends to study a total of 288 patients. The results are being presented before reaching the total sample due to the postgraduate deadlines. The present study was partly funded by the Coordenação de Aperfeiçoamento de Pessoal de Nível Superior-Brasil (Finance Code 001).

### Inclusion Criteria

The inclusion criteria were age between 40 and 65 years old, 1 year of amenorrhea and follicle stimulating hormone (FSH) ≥30 mUI/mL, BMI ≥18.5 kg/m^2^, and sexually active state (having regular sexual encounters with a fixed partner without sexual dysfunction within the last 4 weeks). Women using estrogens and progesterone were included if they took a stable dose for 6 months before screening.

### Exclusion Criteria

A partner with sexual problems such as erectile dysfunction or premature ejaculation, any other psychiatric disorder that can affect sexual function (e.g., schizophrenia, bipolar affective disorder, borderline disorder, anxiety disorder, panic disorder); a history of diagnosed depression; score > 14 on the Beck Depression Inventory; women in Tibolone therapy; use of any drug that, in the opinion of the investigator, can affect sexual function, a history of bilateral oophorectomy; or a cancer diagnosis (cancer of the breast, cervix, endometrium, ovary, intestine, etc.) were excluded.

Obesity was diagnosed according to BMI. Body weight was measured using an electronic scale (accurate to the nearest 0.1 kg) in women with an empty bladder and wearing only underwear. Height was measured to the nearest 0.5 cm, using a wall-mounted stadiometer, in women without footwear. Thus, BMI was calculated according to the World Health Organization (WHO) recommendations for assessing nutritional status^15^ and divided into the following categories: BMI ≤18.5 kg/m^2^, underweight; 18.5–24.9 kg/m^2^, normal weight; 25–29.9 kg/m^2^, overweight; 30–34.9 kg/m^2^, class 1 obesity; 35–39.9 kg/m^2^, class 2 obesity; and ≥40 kg/m^2^, class 3 obesity. In the present study, women were stratified into 3 groups according to BMI: group 1, BMI 18.5–24.9 kg/m^2^ (normal weight); group 2, 25–29.9 kg/m^2^ (overweight); and group 3, BMI ≥30 kg/m^2^ (obese). We hypothesized that the obesity grade is related to sexual dysfunction in postmenopausal women. All participants signed a Free and Informed Consent Form. Sexual function was assessed using the Female Sexual Function Index (FSFI),^16^ a questionnaire validated for Brazilian Portuguese,^17^ with 19 items measuring female sexual function. Cutoff points of ≥23 and ≥26.5 were adopted to define a diagnosis of FSD based on the Diagnostic and Statistical Manual of Mental Disorders, 4^th^ edition, Text Revision by the American Psychiatric Association for a duration of ≥24 weeks.^18^ The score of 23 points was compared with that of 26.5 points to avoid possible biases related to the study of sexual function in Latin American women, who tend to report lower scores for their sexual function in questionnaire surveys, as suggested by Silva et al.^19^ We believe that a less rigorous cutoff value is more appropriate for the Brazilian population, considering that the percentage of the population affected by FSD is very high and incompatible with what we have observed during the interviews.

A score of ≤5 on the combination of items comprising the desire domain of the FSFI questionnaire was used to define the diagnosis of hypoactive sexual desire disorder (HSDD) in postmenopausal women. The questions were as follows: “Over the past 4 weeks, how often did you feel sexual desire or interest?” [5, almost always or always; 4, most times (more than half the time); 3, sometimes (about half the time); 2, a few times (less than half the time); and 1, almost never or never], and “Over the past 4 weeks, how would you rate your level (degree) of sexual desire or interest?” [5, very high; 4, high; 3, moderate; 2, low; and 1, very low or none at all]. A score of ≥6 means the absence of HSDD.^20^

The diagnosis of sexual dysfunction was established by a sexologist (Silva G. M. D.) experienced and trained in diagnosing FSD using the FSFI. The women provided their responses in a private room. We believe that having an experienced evaluator apply the questionnaires increases the reliability of the results. We did not evaluate sexual orientation or gender identity as a criterion for excluding participants. However, all surveyed women declared themselves as heterosexual with a female gender identity. The Beck Depression Inventory^21^ was used to exclude depression in patients with a history of the disease (score > 14).

All women underwent a semistructured interview developed and routinely used in the service with questions such as age, education, marital status, religion, race, personal background and medication use. Blood pressure and waist circumference (WC) were measured; BMI was calculated; gynecological examinations were conducted; and samples for Papanicolaou smears were collected. Subsequently, laboratory examinations (total cholesterol and fractions, triglycerides, and fasting glucose) were ordered, along with bilateral mammography and transvaginal ultrasound.

The study was performed in compliance with the protocol and principles established in the Declaration of Helsinki (1996 version), the International Conference on Harmonization Harmonized Tripartite Guideline for Good Clinical Practice, and applicable regulatory requirements. The protocol was approved by the research ethics committees of the School of Medical Sciences of the Hospital Santa Casa de Misericórdia de São Paulo and of the Hospital Maternidade Leonor Mendes de Barros (CAAE Permit 40594814.4.0000.5479) and registered under Clinical Trials ID NCT02430987.

### Statistical Analysis

The characteristics of the study participants are expressed as absolute and relative frequencies for qualitative measures, whereas summary measures (mean, standard deviations [SDs], median, minimum and maximum) were employed for quantitative measures. The domains of the questionnaire with the preestablished criteria were described according to BMI, and the association was verified using the chi-squared test or the Fisher exact test.^22^ ^23^ Generalized linear models with binomial distribution and logit function were created to compare the presence of sexual dysfunction according to the interest categories adjusted for age, education, race/color, marital status, and religion. The BMI categories were compared using Bonferroni multiple comparisons. The diagnostic components of sexual dysfunction in the FSFI questionnaire were described separately according to BMI. Associations were determined using the chi-squared test or the Fisher exact test. For BMI categories, comparisons among categories were followed by Bonferroni multiple comparisons.^24^

The FSFI scores by BMI category are depicted as box plots. All of the analyses were performed using IBM SPSS Statistics for Windows, Version 20.0 (IBM Corp., Armonk, NY, USA), and tables were produced using StatistiXL Package (Statistical Power for Microsoft Excel version 1.8). A significance level of 5% was adopted for the tests.

The domain and total scores on the FSFI were described according to BMI category. The categories were compared using the Kruskal-Wallis test,^25^ followed by the Dunn multiple comparisons test^24^ in the event of statistical significance.^25^

Sample size was calculated on the basis of the following: one confidence level (1-α): 95; power (% probability of detection): 80; ratio of controls per case: 1; hypothetical proportion of controls with exposure: 40; hypothetical proportion of cases with exposure: 57.14; least extreme odds ratios (ORs) to be detected: 2.00; sample size: 144 cases (obese and overweight) and 144 controls for a total of 288 patients.^24^

## Results

The baseline characteristics of all 221 women are summarized in Table 1. The mean baseline age was 54.3 ± 5 years old. About 65% were living with a spouse or a partner. One-half of the participants were Catholic and self-identified as white. Almost one-fifth of the women had at least a college education. The average BMI was 28.2 kg/m^2^, the mean abdominal circumference was 96.6 cm, the mean weight was 69.8 kg, and the mean height was 1.58 m. The mean total cholesterol was 215.1 mg/dL, the mean HDL was 52.3 mg/dL, the median triglyceride was 157.9 mg/dL, and the median blood glucose was 101.8 mg/dL. A total of 115 (52%) of women had systemic arterial hypertension, 120 (54.3%) met the MetS criteria, and the median total FSFI score was 24.1.

**Table 1:** Patient characteristics

Variable	*Value (n = 221)*
Age (years), average ± SD	54.3 ± 5
Weight (kg), average ± SD	69.8 ± 12.6
Height (m), average ± SD	1.58 ± 0,07
BMI (kg/m^2^), average ± SD	28.2 ± 5.5
AC, average ± SD	96.6 ± 11.3
TC, average ± SD	215.1 ± 38.9
HDL, average ± SD	52.3 ± 10.7
Triglycerides, average ± SD	157.9 ± 98.6
Blood glucose, average ± SD	101.8 ± 34
SAH, n (%)	115 (52)
MetS, n (%)	120 (54.3)
Educational level, *n* (%)	
Illiterate	1 (0.4)
Elementary/middle school	80 (36.2)
High school	95 (43.0)
College	45 (20.4)
Religion, n (%)	
Catholic	116 (52.5)
Evangelical/Protestant	69 (31.2)
Spiritism	10 (4.5)
Umbanda/Candomblé	4 (1.8)
Jehovah's Witness	4 (1.8)
Agnostic	18 (8.1)
Race, *n* (%)	
White	124 (56.1)
Brown	60 (27.1)
Black	37 (16.7)
Marital status, *n* (%)	
Married	144 (65.2)
Single	51 (23.1)
Divorced	22 (10.0)
Widow	4 (1.8)
Desire, median (min; max)	3 (1.2; 6)
Arousal, median (min; max)	3.6 (1.8; 6)
Lubrication, median (min; max)	3.6 (1.2; 6)
Orgasm, median (min; max)	4 (1.2; 6)
Satisfaction, median (min; max)	4.4 (2; 6)
Pain, median (min; max)	6 (1; 6)
Total FSFI score, median (min; max)	24.2 (13.4; 34.8)

Abbreviations: BMI, body mass index; FSFI, Female Sexual Function Index; HDL, high-density lipoprotein; MetS, metabolic syndrome; SAH, systemic arterial hypertension; SD, standard deviation; TC, total cholesterol; WC, waist circumference.

Table 2 shows that the scores for desire, arousal, satisfaction, and total FSFI statistically differed among BMI categories (*p* < 0.05). The presence of an HSDD diagnosis was associated with BMI categories (*p* = 0.003).

**Table 2:** Comparisons of female sexual function index items between groups stratified according to body mass index

	BMI			
Variable	Normal	Overweight	Obese	*p-value*
	(*n* = 55)	(*n* = 98)	(*n* = 68)	
Desire, median (min; max)	3.6 (1.2; 6)	3 (1.2; 5.4)	3 (1.2; 5.4)	0.006
Arousal, median (min; max)	3.6 (2.1; 5.7)	3.6 (2.1; 5.4)	3.45 (1.8; 6)	0.014
Lubrication, median (min; max)	4.2 (1.2; 6)	4.2 (1.2; 6)	3.6 (1.8; 6)	0.080
Orgasm, median (min; max)	4.4 (1.2; 6)	4 (1.2; 6)	4 (1.2; 6)	0.105
Satisfaction, median (min; max)	4.8 (3.2; 6)	4.4 (2; 6)	4.4 (2; 6)	0.003
Pain, median (min; max)	6 (2; 6)	6 (1; 6)	6 (2; 6)	0.967
FSFI score, median (min; max)	25 (15.1; 34.8)	24.2 (13.6; 32.5)	23.8 (13.4; 32.3)	0.027
FSD-cutoff ≥23 points, *n* (%)	18 (32.7)	40 (40.8)	33 (48.5)	0.208*
FSD-cutoff ≥26.5 points, *n* (%)	32 (58.2)	65 (66.3)	50 (73.5)	0.200*
HSDD diagnosis, *n* (%)	20 (36.4)	56 (57.1)	45 (66.2)	0.003*

Abbreviations: BMI, body mass index; FSFI, Female Sexual Function Index; FSD, female sexual function; HSDD, hypoactive sexual desire disorder.

Kruskal-Wallis test.

* Chi-squared test.

Table 3 shows that the desire and arousal scores were statistically higher in women in the normal BMI group than in those in the obese group (*p* = 0.028 and *p* = 0.043, respectively).

**Table 3:** Results of multiple comparisons of body mass index categories for domains and sexual dysfunction exhibiting statistically significant differences

Variable	Comparison		*z-value*	*p-value*
Desire	Normal versus	Overweight	1.59	0.113
	Normal versus	Obese	2.20	0.028
	Overweight versus	Obese	0.84	0.402
Arousal	Normal versus	Overweight	1.54	0.124
	Normal versus	Obese	2.03	0.043
	Overweight versus	Obese	0.69	0.491
Satisfaction	Normal versus	Overweight	2.21	0.027
	Normal versus	Obese	2.00	0.045
	Overweight versus	Obese	− 0.06	0.955
FSFI total score	Normal versus	Overweight	1.38	0.169
	Normal versus	Obese	1.88	0.060
	Overweight versus	Obese	0.69	0.492
HSDD diagnosis (%)*	Normal versus	Overweight	− 21.0	0.034
	Normal versus	Obese	− 30.0	0.002
	Overweight versus	Obese	− 9.0	0.706

Abbreviations: FSFI, Female Sexual Function Index; HSDD, hypoactive sexual desire disorder.

Dunn multiple comparisons.

* Bonferroni multiple comparisons.

**Fig. 2:**
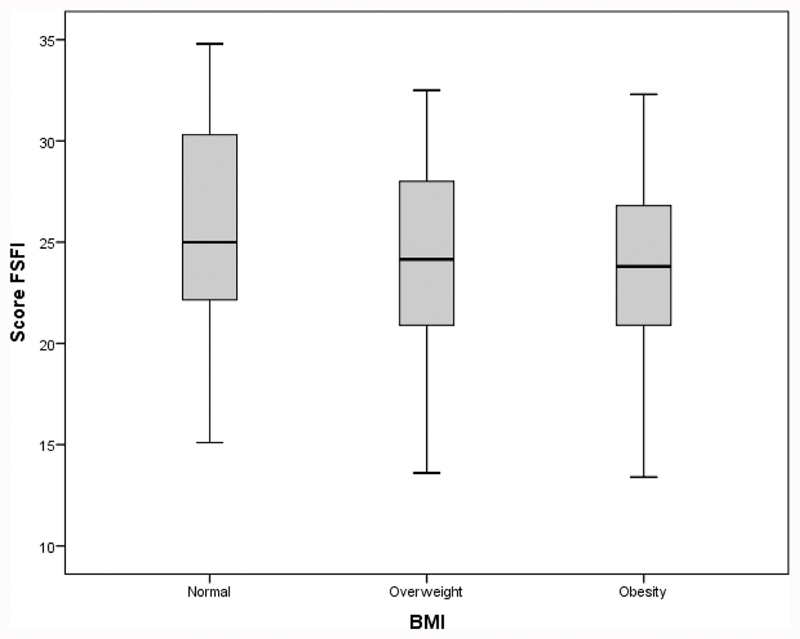
Box plots of the Female Sexual Function Index (FSFI) scores by BMI category. Abbreviations: BMI, body mass index.

The satisfaction score was statistically higher in the normal BMI group than in the overweight and obese groups (*p* < 0.05). The incidence of HSDD diagnosis was statistically lower in the normal BMI group than in the overweight and obese groups (*p* = 0.034 and *p* = 0.002, respectively). The total FSFI score statistically differed across BMI categories (Table 2, p = 0.027); however, it was not possible to identify through the multiple comparisons between which BMI categories this difference occurred (p > 0.05). Nevertheless, the results suggested lower scores in obese women than in women with normal BMI (*p* = 0.060). Fig. 2 depicts the results from Tables 2 and 3, showing slightly higher total FSFI scores in women with a normal BMI than in those in the other categories, especially obese women.

## Discussion

Few studies have explored the relationship between obesity and female sexual function, particularly in postmenopausal women. The issue of health of climacteric women is important not only because of the occurrence of uncomfortable symptoms that alter their quality of life, but also because of its impact on public health owing to the high prevalence of serious diseases such as MetS and obesity. A clearer understanding of this relationship can change the clinical approach with respect to the impact of chronic diseases on female sexuality. Therefore, in the present study, we investigated whether obesity is a risk factor for sexual dysfunction in postmenopausal women.

Both overweight and obesity have been identified as risk factors for sexual dysfunction in men^26^; however, their association with female sexual function remains unclear.^27^ Few studies have addressed sexuality in obese postmenopausal women.^28^ ^29^ ^30^ In fact, the association between sexual function and obesity in women during menopause has become a focus of investigations in recent years, and most studies have shown that obesity is related to low sexual function scores.^28^ ^29^ ^30^ In support of these conclusions, the present study demonstrates higher sexual dysfunction indexes in obese postmenopausal women.

In one of the first studies on the subject,^28^ women with FSD were stratified according to BMI into normal weight (BMI < 25 kg/m^2^) or overweight/obese (BMI ≥25 kg/m^2^) groups. The total FSFI score was significantly lower in overweight/obese women. The FSFI scores strongly correlated with BMI (r = - 0.72, *p* = 0.0001) in women with FSD, as also found in our study. According to Esposito et al,^28^ of the 6 sexual function domains, only desire (r = 0.24, *p* = 0.08) and pain (r = - 0.138, *p* = 0.3) were not correlated with BMI.

In the present study, BMI also showed no correlation with the pain domain, but a statistically significant association with the desire domain was detected. Obese women had a higher prevalence of HSDD than normal-weight women. In the study by Esposito et al,^28^ desire was the only domain exhibiting a positive, albeit non-significant, relationship with BMI. This led to the hypothesis that the female sexual function domains (desire, arousal, lubrication, and orgasm) may not have a linear progression.^28^ In the present study, the sexual desire domain score of the FSFI was lower in overweight and obese women, whereas the number of women with HSDD in these groups was higher than that in the normal-weight group. We did not address the presence or absence of vaginal or anal penetration, and this can pose a bias related to the pain domain. Nevertheless, we excluded women with a history of pelvic surgery.

However, there was a strong negative correlation between BMI and arousal (r = - 0.75, *p* < 0.001), lubrication (r = - 0.66, *p* < 0.001), orgasm (r = - 0.56, *p* < 0.001), and satisfaction (r = - 0.56, *p* < 0.001).^28^ Obese women had statistically lower indices of arousal and sexual satisfaction. Concerning the orgasm domain, no significant differences were detected, in contrast to the findings of Esposito et al.^28^ In the same study, no correlation between the total FSFI score and BMI was found among women without FSD. The absence of a relationship between BMI and FSFI in women without FSD (r = 0.2, *p* = 0.09) suggests that obesity may be an important factor when FSD emerges; however, further prospective studies are needed to elucidate this issue.^28^

In the Women's Health Across the Nation (SWAN) study,^29^ a prospective longitudinal multiethnic cohort study in postmenopausal women conducted in the United States, sexual function variables were measured using a 20-item self-reported questionnaire collecting data on sexual function in menopausal women. The analyses included a total of 2,528 women (mean age, 46 years old; mean BMI, 27.7 kg/m^2^) followed-up for an average period of 8 years. Despite the large number of women studied, we believe that self-reported questionnaires are associated with greater bias in responses owing to difficulties in understanding the questions. Therefore, the questionnaires in the present study were applied by a trained sexologist. In addition, the prospective design of the study can help eliminate the cause-effect bias inherent to cross-sectional studies.

In the SWAN study, all of the sexual function variables significantly declined over time (i.e., desire, frequency of sexual intercourse, and ability to attain orgasm, *p* < 0.001; arousal, *p* = 0.001). Body mass index also significantly increased over time (*p* < 0.001) with a mean BMI of 29.1 kg/m^2^ at the study endpoint. Global changes in desire, arousal, frequency of sexual intercourse, and ability to attain orgasm were not associated with general changes in BMI during the course of the study.

Deviations from the expected BMI of each participant on a given year as a function of individual evolution proved a predictor of change in sexual functioning. The frequency of sexual intercourse was significantly associated with deviations from the expected evolution of BMI in each woman, where greater deviations below (or above) the expected evolution were associated with a lower (or greater) frequency of sexual intercourse. Similarly, with deviations in BMI above or below the expected evolution for a given year, the participants reported lower or higher-than-expected levels of desire, respectively (*p* = 0.020). Deviation from the expected BMI evolution at each time point was not associated with level of arousal or ability to attain orgasm.

In the present study, a higher BMI was associated with worse sexual function indices, particularly sexual desire and arousal, and the differences were statistically significant. As in the SWAN study, no changes in orgasm with increased BMI were found. Although the FSFI does not directly assess the frequency of sexual activity, the instrument includes sexual satisfaction, which may serve as a proxy because sexual dissatisfaction can lead to sexual avoidance. The study results showed that postmenopausal women with a higher BMI had a greater level of sexual dissatisfaction.

In a cross-sectional study, Simoncig Netjasov et al^30^ investigated the hormonal profile of 73 postmenopausal women (age range, 50–65 years old). The participants were divided into obese subjects (mean BMI, 35.9 kg/m^2^) and non-obese controls (mean BMI, 22.5 kg/m^2^), all of whom completed the McCoy Female Sexuality Questionnaire. The results showed that obese women had less pain during sexual intercourse but reported less pleasurable intercourse with lower arousal and fewer orgasms than the controls and tended to be less satisfied with their partner. Although not associated with the pain score, obese women had statistically lower indices of desire, arousal, and sexual satisfaction. No significant differences in the orgasm domain were detected. Obese and overweight women had a higher prevalence of HSDD than normal-weight women. In contrast to Simoncig Netjasov et al,^30^ who assessed different components of sexual response, we used the FSFI questionnaire. Nevertheless, our study corroborated their results by concluding that obesity has an important impact on the sexual function of postmenopausal women.

In the study of Mostafa et al,^31^ findings showed a negative association between BMI and FSFI; this correlation was statistically insignificant (*p* = 0.078). A total of 150 overweight and obese women participated in the study. The age of the women ranged between 20 and 49 years old with a mean age of 31.2 ± 7.3 years old. Of them, 133 (88.7%) were overweight and 17 (11.3%) were obese, and the mean BMI of all women stood at 27.5 ± 1.9 kg/m2. We studied postmenopausal women with an average BMI of 28.2 kg/m^2^.

Some limitations of our study should be recognized. First, its transverse nature does not allow inferring a cause-effect correlation. Second, the sample size was small. Finally, as in most epidemiological studies, there is potential for confusion due to the presence of uncontrolled covariates. Moreover, the analyzed women were recruited from among those attending our outpatient clinic and do not necessarily represent the general population.

Human sexuality is a complex phenomenon involving both psychological and organic processes that vary over time; thus, it is highly difficult to study. In addition to the methodological problems encountered by most investigators, there is difficulty in interpreting and comparing published results on this aspect of human behavior. Importantly, studies correlating sexual function and female obesity remain scarce. Nevertheless, the existing studies seem to confirm that a higher BMI is correlated with more sexual dysfunction and that weight loss leads to improved sexual function.

## Conclusion

Our results show that obese and overweight postmenopausal women had a higher index of dysfunction in desire and arousal and lower sexual satisfaction than normal-weight women. However, no relationship with orgasm or pain scores was found.
